# Protective Effect of Remote Ischemic Preconditioning against Myocardial Ischemia-Reperfusion Injury in Rats and Mice: A Systematic Review and Meta-Analysis

**DOI:** 10.31083/j.rcm2312413

**Published:** 2022-12-20

**Authors:** Lu Chen, Yan Weng, Ailing Qing, Jun Li, Pingliang Yang, Ling Ye, Tao Zhu

**Affiliations:** ^1^Department of Anesthesiology, West China Hospital, Sichuan University, 610041 Chengdu, Sichuan, China; ^2^Department of Anesthesiology, The People's Hospital of Jianyang, 641400 Jianyang, Sichuan, China; ^3^Department of Anesthesiology, West China School of Public Health and West China Fourth Hospital, Sichuan University, 610041 Chengdu, Sichuan, China; ^4^Department of Pain Management, West China Hospital, Sichuan University, 610041 Chengdu, Sichuan, China; ^5^Department of Anesthesiology, The First Affiliated Hospital of Chengdu Medical College, 610500 Chengdu, Sichuan, China

**Keywords:** remote ischemic preconditioning, myocardial ischemia-reperfusion injury, meta-analysis, systematic review

## Abstract

**Background:**

Remote ischemic preconditioning (RIPC) has cardioprotective effects. This study was designed to evaluate the effectiveness and potential influencing factors of RIPC for myocardial ischemia-reperfusion injury (MIRI) in rats and mice.

**Methods:**

The PubMed, Web of Science, Embase, and Cochrane Library databases were searched to identify animal model studies that explored the effect of RIPC on MIRI. The primary outcome was myocardial infarct size, and secondary outcomes included serum cardiac markers, vital signs, hemodynamic parameters, and TUNEL-positive cells. Quality was assessed using SYRCLE’s Risk of Bias Tool.

**Results:**

This systematic review and meta-analysis included 713 male animals from 37 studies. RIPC significantly protected against MIRI in small animal models by reducing infarct size, decreasing serum myocardial marker levels and cell death, and improving cardiac function. Subgroup analysis indicated that RIPC duration and sites influence the protective effect of RIPC on MIRI. Meta-regression suggested that study type and staining method might be sources of heterogeneity. The funnel plot, Egger’s test, and Begg’s test suggested the existence of publication bias, but results of the sensitivity analysis and nonparametric trim-and-fill method showed that the overall effect of RIPC on MIRI infarct size was robust.

**Conclusions:**

RIPC significantly protected against MIRI in small animal models by reducing infarct size, decreasing serum myocardial markers and limiting cell death, and improving cardiac function. RIPC duration and site influence the protective effect of RIPC on MIRI, which contributes in reducing confounding factors and determines the best approach for human studies.

## 1. Introduction 

Cardiovascular diseases are major contributors to the disease burden, with acute myocardial infarction being the most severe manifestation. Disability-adjusted life years due to ischemic heart disease have reached 182 million, which increased steadily from 1990 to 2019, causing global mortality and rise in healthcare costs [1]. Reperfusion and revascularization strategies, such as percutaneous coronary intervention and coronary artery bypass grafting, have been widely used to improve perfusion and prevent acute re-occlusion in acute myocardial infarction [2]. However, reperfusion could also lead to additional injury, including increased infarct size and microvascular dysfunction [3]. This is called myocardial ischemia-reperfusion injury (MIRI). MIRI is generally related to calcium overload, increased reactive oxygen species, proinflammatory factors, endoplasmic reticulum stress, and mitochondrial dysfunction [4], leading to different forms of cell death [5].

Murry first reported ischemic preconditioning in 1986 as a non-pharmacological intervention for MIRI [6]. Remote ischemic preconditioning (RIPC), brief and transient episodes of ischemia at a remote site before myocardial ischemia, have been reported to have a cardioprotective effect [7]. Some clinical experiments have explored the cardioprotective effect of RIPC in patients undergoing surgery; the results were controversial, which might be attributed to confounding factors, such as age, comorbidities, surgery, anesthesia, medication, and RIPC method [8, 9, 10, 11, 12]. Therefore, finding an ideal protocol, including the appropriate site, duration, and cycles of RIPC, and exploring the factors influencing its protective effect from preclinical evidence are important for optimizing RIPC in clinical studies. Although multiple animal studies have been conducted to explore the effect and mechanism of RIPC on MIRI, there is still a lack of systematic reviews and meta-analyses to assess the overall effect of RIPC on MIRI in small animal studies. Therefore, this study was designed to evaluate the effects and potential influencing factors of RIPC on MIRI in rats and mice.

## 2. Materials and Methods

### 2.1 Search Strategys

This systematic review and meta-analysis was performed according to the PRISMA guidelines and registered in PROSPERO (CRD42022362017). Four databases, including PubMed, Web of Science, Embase, and Cochrane Library, were searched until June 13, 2022, to identify animal studies exploring the effect of RIPC on MIRI. The search keywords were “Remote ischemic preconditioning”, “RIPC”, “myocardial ischemia-reperfusion injury”, “MIRI”, “MIR”, and “myocardial reperfusion injury”.

### 2.2 Inclusion and Exclusion Criteria

The inclusion criteria were as follows: (1) studies on young male rats or mice; (2) studies using *in vivo* or *ex vivo* models of MIRI; (3) where animals in the treatment group received RIPC, while the control group received a placebo or no treatment; (4) myocardial infarction size was measured by triphenyl tetrazolium chloride (TTC) staining and reported as a percentage; and (5) language limited to English. The exclusion criteria were as follows: (1) studies on animals with other comorbidities such as diabetes or hyperlipidemia; (2) having incomplete data; (3) duplicate publications; (4) review, conference abstract, comment, and protocol; and (5) studies where animals received substances obtained from the blood of humans who received RIPC.

### 2.3 Data Extraction

After removing duplicates, two authors screened for eligibility by browsing the titles and abstracts of the records, followed by the full text. Another author was consulted in case of any disagreement. After confirming the included studies, two authors extracted the data independently using Excel 2019 (Microsoft Corp., Redmond, WA, USA), and disagreements were resolved by another author. Study characteristics were extracted, including author names, year of publication, country, species, animal weight, number of animals in both groups, anesthesia method, ischemia/reperfusion (I/R) method, I/R duration, RIPC protocol, RIPC site, outcome measurement, and staining method. The primary outcome was myocardial infarct size, and secondary outcomes included serum cardiac markers, vital signs, hemodynamic parameters, and TUNEL-positive cells. If there were any missing data or the data were presented in figures, the corresponding author would be contacted for more information. Data, including the number, mean, and standard deviation (SD), were collected. If there was only a standard error of the mean (SEM) reported in the articles, SEM was transformed into SD.

### 2.4 Quality Assessment

Two authors evaluated the quality of the included studies using SYRCLE’s Risk of Bias Tool [13] which is recommended for animal studies. A third author was invited to resolve any disagreements. This tool assessed six domains of bias: selection bias (sequence generation, baseline characteristics, and allocation concealment), performance bias (random housing and blinding), detection bias (random outcome assessment and blinding), attrition bias (incomplete outcome data), reporting bias (selective outcome reporting), and other sources of bias. Each entry was evaluated as unclear, low risk, or high risk. The higher the score, the better the quality of the study.

### 2.5 Statistical Analysis

Stata version 12.0 (Stata Corp., College Station, TX, USA) was used for the meta-analysis. The standardized mean difference with a 95% confidence interval (95% CI) was used to evaluate the difference between the RIPC and control groups. Heterogeneity was evaluated using Cochran’s Q test and Higgins’ $I^{2}$ statistic. The random-effects model was applied for the pooled effect estimates if *p *< 0.10 and/or $I^{2}$ > 50%; otherwise, the fixed-effects model was used. Subgroup and meta-regression analyses were used to explore sources of heterogeneity. We performed a sensitivity analysis by excluding each study to assess its impact on the results. Publication bias was evaluated using funnel plots, Egger’s test, and Begg’s test.

## 3. Results

### 3.1 Study Selection

**Fig. 1.**
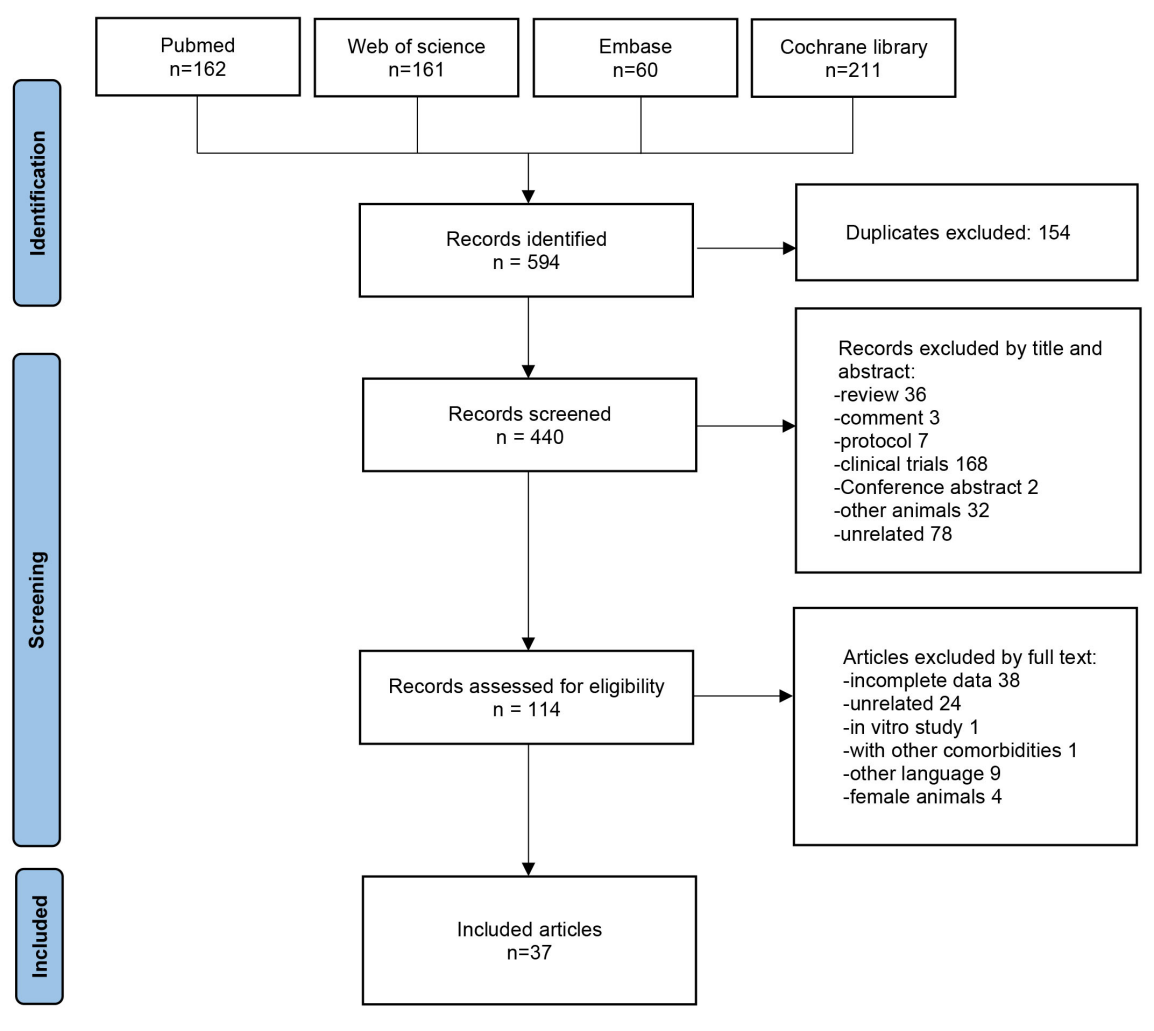
**Prisma chart flow**. A total of 594 studies were identified from Pubmed, Web of Science, Embase, and Cochrane library. After assessed of eligibility, 37 articles were included in the quantitative synthesis.

A total of 594 studies were identified from PubMed, Web of Science, Embase, and Cochrane Library databases. After removing duplicates, 440 studies underwent title and abstract screening, and 326 were excluded. The full texts of the remaining 114 studies were assessed. Seventy-seven studies were excluded; 38 owing to incomplete data, 24 owing to unrelated data, 1 was an *in vitro* study, 1 reported on other comorbidities, 9 were in another language, and 4 were on female animals. Finally, 37 articles were included in the quantitative synthesis [14, 15, 16, 17, 18, 19, 20, 21, 22, 23, 24, 25, 26, 27, 28, 29, 30, 31, 32, 33, 34, 35, 36, 37, 38, 39, 40, 41, 42, 43, 44, 45, 46, 47, 48, 49, 50] (Fig. 1).

### 3.2 Study Characteristics

The meta-analysis included 37 published articles from which we extracted data of 45 comparative studies between RIPC and control groups in MIRI models, and the characteristics of the articles are shown in Table 1 (Ref. [14, 15, 16, 17, 18, 19, 20, 21, 22, 23, 24, 25, 26, 27, 28, 29, 30, 31, 32, 33, 34, 35, 36, 37, 38, 39, 40, 41, 42, 43, 44, 45, 46, 47, 48, 49, 50]). These articles were published in China, Germany, France, Australia, India, Argentina, America, Japan, Slovakia, Denmark, Spain, the Netherlands, the Russian Federation, and the United Kingdom from 2005 to 2021. A total of 27 articles used the *in vivo* MIRI model by ligation and release of the left anterior descending coronary artery, and the remaining 10 used the *ex vivo* MIRI model by Langendorff perfusion. A total of 713 animals were included in this meta-analysis, including Friend Virus Bmice, C57Bl/6 mice, Wistar rats, Sprague–Dawley rats, and Lewis rats. The RIPC site included the limb, femoral artery, infrarenal aorta, mesenteric artery, hepatic vessels, and abdominal aorta.

**Table 1.** **Characteristics of the included studies**.

Author	Year	Country	Species	Weight	RIPC/control	Anesthesia	I/R method	I/R duration	RIPC site	RIPC protocol	Outcome measurements	Staining method
Ren [14]	2021	China	FVB mice	unclear	6/6	50 mg/kg thiopental	*ex vivo*	30/90 min	hepatic occlusion of the portal triad	5/5 min 3 cycles	infarct size, CK-MB, LDH, HR, LVDP	TTC
Lucia [15]	2021	Slovakia	Wistar rats	250 ± 10 g	8/8	50–60 mg/kg thiopental	*ex vivo*	30/120 min	right hind limb by pressure cuff (200 mmHg)	5/5 min 3 cycles	infarct size, recovery of LVDP, duration of VT	TTC
Marie [16]	2021	Denmark	SD rats	300 g	8/7	65 mg/kg pentobarbital	*ex vivo*	30/120 min	right hind limb (tourniquet)	5/5 min 3 cycles	infarct size, HR, LVDP, RPP, CF, LDH	TTC
Yasuaki [17]	2020	Japan	Wistar rats	unclear	8/8	2 mg/kg midazolam, 2.5 mg/kg butorphanol and 0.15 mg/kg medetomidine	*in vivo*	30/120 min	right forelimb and hindlimb	5/5 min 3 cycles	infarct size, HR, MAP	TTC-Evans blue
Ke [18]	2020	China	SD rats	250–300 g	6/6	50 mg/kg pentobarbital	*in vivo*	30/120 min	unilateral hindlimb	5/5 min 3 cycles	infarct size, HR, MAP, RPP	TTC-Patent blue
Billah [19]	2020	Australia	SD rats	300–350 g	8/8	2–5% isoflurane	*in vivo*	30 min/24 h	hindlimb	5/5 min 3 cycles	infarct size	TTC-Evans blue
Diamela [20]	2019	Argentina	SD rats	200–250 g	6/6	65 mg/kg urethane	*ex vivo*	30/120 min	femoral artery occlusion	5/5 min 3 cycles	infarct size	TTC
Billah [21]	2019	Australia	SD rats	300–350 g	8/10	2–5% isoflurane	*in vivo*	30 min/24 h	hindlimb	5/5 min 3 cycles	infarct size	TTC-Evans blue
Sapna [22]	2019	India	Wistar rats	150–220 g	6/6	50 mg/kg thiopental	*ex vivo*	30/120 min	hindlimb	5/5 min 4 cycles	infarct size, LDH, CK, LVDP, dp/dt max, dp/dt min	TTC
Patrick [23]	2018	Germany	Wistar rats	293 ± 22 g	9/10	100 mg/kg pentobarbital	*in vivo*	25/120 min	bilateral hind-limb ischemia by blood pressure cuff (200 mmHg)	5/5 min 4 cycles	infarct size, HR, MAP	TTC-Evans blue
Xavier [24]	2018	Spain	C57Bl/6 mice	unclear	16/19	60 mg/kg pentobarbital	*in vivo*	40/120 min	right hindlimb vascular occlusion	5/5 min 3 cycles	infarct size	TTC-Evans blue
Helmut [25]	2018	Germany	Lewis rats	200–380 g	16/8	100 mg per 10 mg/kg ketamine/xylazine	*ex vivo*	30/120 min	left hindlimb	5/5 min 3 cycles	infarct size	TTC-Patent blue
Chen [26]	2018	China	Mice	unclear	8/7	2–5% isoflurane	*in vivo*	30/180 min	left femoral artery occlusion	5/5 min 3 cycles	infarct size, TUNEL-positive cells, CK, CK-MB, LDH	TTC-Evans blue
Friederike [27]	2018	Netherlands	Wistar rats	301 ± 17 g	6/6	40 mg/kg/h pentobarbital	*in vivo*	25/120 min	bilateral hind-limb	5/5 min 4 cycles	infarct size, HR, MAP	TTC-Evans blue
					6/6	Sevoflurane (1 minimal alveolar concentration) + remifentanil (0.5 µg/kg/min)						
					6/6	Propofol (12 mg/kg/h) + remifentanil (0.5 µg/kg/min)						
Yu [28]	2017	China	SD rats	250–300 g	10/10	ethyl carbamate	*ex vivo*	30/60 min	unilateral hindlimb	5/5 min 4 cycles	infarct size, HR, LVEDP, RPP, dp/dt max, dp/dt min, cTNI	TTC
Yang [29]	2017	China	SD rats	250–300 g	5/5	50 mg/kg pentobarbital	*in vivo*	45/180 min	hepatic vessel clamp	5/5 min 3 cycles	infarct size, serum LDH and CK-MB, LVSP, LVEDP, dp/dt max, dp/dt min, TUNEL-positive cells	TTC
Amritpal [30]	2017	India	Wistar rats	150–220 g	6/6	50 mg/kg thiopental	*ex vivo*	30/120 min	hindlimb by blood pressure cuff (150 mmHg)	5/5 min 4 cycles	infarct size, LDH, CK, LVDP, dp/dt max, dp/dt min	TTC
Mudaliar [31]	2017	Australia	SD rats	250–300 g	7/7	unclear	*in vivo*	30 min/24 h	hindlimb	5/5 min 3 cycles	infarct size	TTC-Evans blue
Friederike [32]	2017	Germany	Wistar rats	263±18 g	6/6	100 mg/kg pentobarbital	*in vivo*	25/120 min	bilateral hind-limb ischemia by blood pressure cuff (200 mmHg)	5/5 min 4 cycles	infarct size, HR, MAP	TTC
Michael [33]	2016	Russian Federation	Wistar rats	220–260 g	7/7	60 mg/kg pentobarbital	*in vivo*	30/90 min	infrarenal aorta occlusion	5/15 min 1 cycle	infarct size, VT/VF duration, mortality rates, HR, MAP	TTC-Evans blue
					7/7					15/15 min 1 cycle		
					6/7					30/15 min 1 cycle		
					12/14				mesenteric artery occlusion	15/15 min 1 cycle		
					6/10					15/15 min 1 cycle		
Donato [34]	2016	Argentina	Wistar rats	200–250 g	10/8	65 mg/kg pentobarbital	*ex vivo*	30/120 min	left femoral artery occlusion	5/5 min 3 cycles	infarct size	TTC
Juan [35]	2016	France	Wistar rats	unclear	10/8	60 mg/kg pentobarbital	*in vivo*	40/120 min	upper right femoral artery occlusion	5/5 min 4 cycles	infarct size	TTC
Laura [36]	2016	France	Wistar rats	200–250 g	6/6	60 mg/kg pentobarbital	*in vivo*	40/120 min	upper right femoral artery occlusion	5/5 min 4 cycles	infarct size	TTC-Evans blue
Chai [37]	2015	China	SD rats	250–300 g	14/14	50 mg/kg pentobarbital	*in vivo*	30/180 min	bilateral femoral artery occlusion	5/5 min 3 cycles	infarct size, HR, MAP, Serum cTNI, TUNEL-positive cells	TTC-Evans blue
Tienush [38]	2014	Germany	C57Bl/6 mice	32 ± 6 g	5/5	45 mg/kg ketamine and 10 mg/kg xylazine	*in vivo*	30 min/24 h	right upper hindlimb	5/5 min 4 cycles	infarct size	TTC-Evans blue
Hussein [39]	2014	France	Wistar rats	200–250 g	6/7	60 mg/kg pentobarbital	*in vivo*	40/120 min	upper right femoral artery occlusion	5/5 min 4 cycles	infarct size	TTC-Evans blue
Chai [40]	2014	China	SD rats	250–300 g	15/15	50 mg/kg pentobarbital	*in vivo*	30/180 min	bilateral femoral arteries occlusion	5/5 min 3 cycles	infarct size, HR, MAP, serum CK-MB, cTNI, LDH, TUNEL-positive cells	TTC-Evans blue
					15/15				abdominal aorta occlusion			
Timo [41]	2014 (1)	Germany	Wistar rats	unclear	6/6	100 mg/kg pentobarbital	*in vivo*	35/120 min	bilateral hind limb	5/5 min 4 cycles	infarct size, HR, MAP	TTC-Evans blue
Timo [42]	2014 (2)	Germany	Wistar rats	unclear	6/6	80 mg/kg pentobarbital	*in vivo*	35/120 min	bilateral hind limb	5/5 min 4 cycles	infarct size, HR, MAP	TTC-Evans blue
Zhu [43]	2013	China	Wistar rats	340 ± 59 g	6/6	3 mL/kg 10% chloral hydrate	*in vivo*	30/180 min	bilateral hind limb	5/5 min 3 cycles	infarct size, arrhythmic score, LVSP, LVEDP, dp/dt max, dp/dt min	TTC-Evans blue
Pierre [44]	2013	France	Wistar rats	unclear	11/9	60 mg/kg pentobarbital	*in vivo*	40/120 min	upper right femoral artery occlusion	10/10 min 1 cycle	infarct size	TTC-Evans blue
Cai [45]	2013	America	Mice	unclear	6/6	70 mg/kg pentobarbital	*in vivo*	30/120 min	left femoral artery occlusion	5/5 min 3 cycles	infarct size	TTC-Evans blue
Duan [47]	2012	China	SD rats	300–350 g	5/5	50 mg/kg pentobarbital	*ex vivo*	30/60 min	bilateral femoral artery occlusion	5/5 min 3 cycles	infarct size, TUNEL-positive cells, HR, LVDP, CF, dp/dt max, dp/dt min	TTC
Lu [46]	2012	China	SD rats	280–300 g	6/6	50 mg/kg pentobarbitone	*in vivo*	30/120 min	right femoral artery by vessel clip	5/5 min 1 cycle	infarct size, HR, MAP, RPP, CF	TTC-Evans blue
					6/6					5/5 min 3 cycles		
Nicole [48]	2011	Germany	Wistar rats	300–350 g	6/6	80 mg/kg pentobarbital	*in vivo*	35/120 min	hindlimb	5/5 min 4 cycles	infarct size, HR, MAP, serum CK and TnT	TTC-Evans blue
Shiang [49]	2010	United Kingdom	C57Bl/6 mice	25–30 g	9/10	0.01 mL/g of 10 mg/mL ketamine, 2 mg/mL xylazine and 0.06 mg/mL atropine	*in vivo*	30/120 min	left femoral artery occlusion	5/5 min 3 cycles	infarct size, HR, MAP	TTC-Evans blue
Sebastian [50]	2005	Germany	Wistar rats	290–350 g	6/6	70 mg/kg pentobarbital	*in vivo*	30/150 min	mesentery artery occlusion	15/15 min 1 cycle	infarct size, MAP	TTC-Black Chinese ink

Abbreviation: RIPC, remote Ischemic preconditioning; I/R, ischemia/reperfusion; TTC, 2,3,5-Triphenyltetrazolium chloride; CK, creatine kinase; LDH, lactate dehydrogenase; cTnI, cardiac troponin I; TnT, troponin T; HR, heart rate; MAP, mean arterial pressure; RPP, heart rate-blood pressure product; LVDP, left ventricular developed pressure; LVSP, left ventricular systolic pressure; LVEDP, left ventricular end diastolic pressure; CF, coronary flow; VT, ventricular tachycardia; VF, ventricular fibrillation.

### 3.3 Quality Assessment 

The SYRCLE Risk of Bias Tool was used to assess the quality of the included studies (Table 2, Ref. [14, 15, 16, 17, 18, 19, 20, 21, 22, 23, 24, 25, 26, 27, 28, 29, 30, 31, 32, 33, 34, 35, 36, 37, 38, 39, 40, 41, 42, 43, 44, 45, 46, 47, 48, 49, 50]). Four studies [33, 37, 40, 43] scored six points, while 10 studies [19, 20, 21, 31, 34, 35, 36, 44, 45, 50] scored only two points. None of the studies described whether allocation concealment was performed, and performance bias (blinding) was at high risk.

**Table 2.** **Quality assessment of included studies**.

Study	A	B	C	D	E	F	G	H	I	J	Total
Ren 2021 [14]	?	Y	?	Y	N	?	?	?	Y	Y	4
Lucia 2021 [15]	?	Y	?	Y	N	?	?	?	Y	Y	4
Marie 2021 [16]	?	Y	?	Y	N	?	Y	?	Y	Y	5
Yasuaki 2020 [17]	?	Y	?	Y	N	?	Y	?	Y	Y	5
Ke 2020 [18]	?	Y	?	?	N	?	?	Y	Y	Y	4
Billah 2020 [19]	?	?	?	?	N	?	?	?	Y	Y	2
Diamela 2019 [20]	?	?	?	?	N	?	?	?	Y	Y	2
Billah 2019 [21]	?	?	?	?	N	?	?	?	Y	Y	2
Sapna 2019 [22]	?	Y	?	Y	N	?	?	?	Y	Y	4
Patrick 2018 [23]	?	Y	?	?	N	?	Y	?	Y	Y	4
Xavier 2018 [24]	?	?	?	?	N	?	?	Y	Y	Y	3
Helmut 2018 [25]	?	Y	?	?	N	?	Y	Y	Y	Y	5
Chen 2018 [26]	?	Y	?	?	N	?	Y	?	Y	Y	4
Friederike 2018 [27]	?	Y	?	?	N	?	Y	?	Y	Y	4
Yu 2017 [28]	?	Y	?	Y	N	?	Y	?	Y	Y	5
Yang 2017 [29]	?	Y	?	Y	N	?	?	Y	Y	Y	5
Amritpal 2017 [30]	?	Y	?	Y	N	?	?	?	Y	Y	4
Mudaliar 2017 [31]	?	?	?	?	N	?	?	?	Y	Y	2
Friederike 2017 [32]	?	Y	?	?	N	?	?	?	Y	Y	3
Michael 2016 [33]	?	Y	?	Y	N	?	Y	Y	Y	Y	6
Donato 2016 [34]	?	?	?	?	N	?	?	?	Y	Y	2
Juan 2016 [35]	?	?	?	?	N	?	?	?	Y	Y	2
Laura 2016 [36]	?	?	?	?	N	?	?	?	Y	Y	2
Chai 2015 [37]	Y	Y	?	Y	N	?	Y	?	Y	Y	6
Tienush 2014 [38]	?	?	?	Y	N	?	?	?	Y	Y	3
Hussein 2014 [39]	?	?	?	?	N	?	Y	?	Y	Y	3
Chai 2014 [40]	Y	Y	?	Y	N	?	Y	?	Y	Y	6
Timo 2014 (1) [41]	?	Y	?	Y	N	?	?	?	Y	Y	4
Timo 2014 (2) [42]	?	Y	?	?	N	?	?	?	Y	Y	3
Zhu 2013 [43]	?	Y	?	?	N	Y	Y	Y	Y	Y	6
Pierre 2013 [44]	?	?	?	?	N	?	?	?	Y	Y	2
Cai 2013 [45]	?	?	?	?	N	?	?	?	Y	Y	2
Duan 2012 [47]	?	Y	?	?	N	?	?	?	Y	Y	3
Lu 2012 [46]	?	Y	?	?	N	?	?	Y	Y	Y	4
Nicole 2011 [48]	?	Y	?	Y	N	?	?	?	Y	Y	4
Shiang 2010 [49]	?	Y	?	?	N	?	?	?	Y	Y	3
Sebastian 2005 [50]	?	?	?	?	N	?	?	?	Y	Y	2

Note: Selection bias: A, Sequence generation; B, Baseline characteristics; C, Allocation concealment. Performance bias: D, Random housing; E, Blinding. Detection bias: F, Random outcome assessment; G, Blinding. Attrition bias: H, Incomplete outcome data. Reporting bias: I, Selective outcome reporting. Other: J, Other sources of bias. ?, unclear; Y, low risk; N, high risk.

### 3.4 Outcome Measures

#### 3.4.1 Infarct Size

**Fig. 2.**
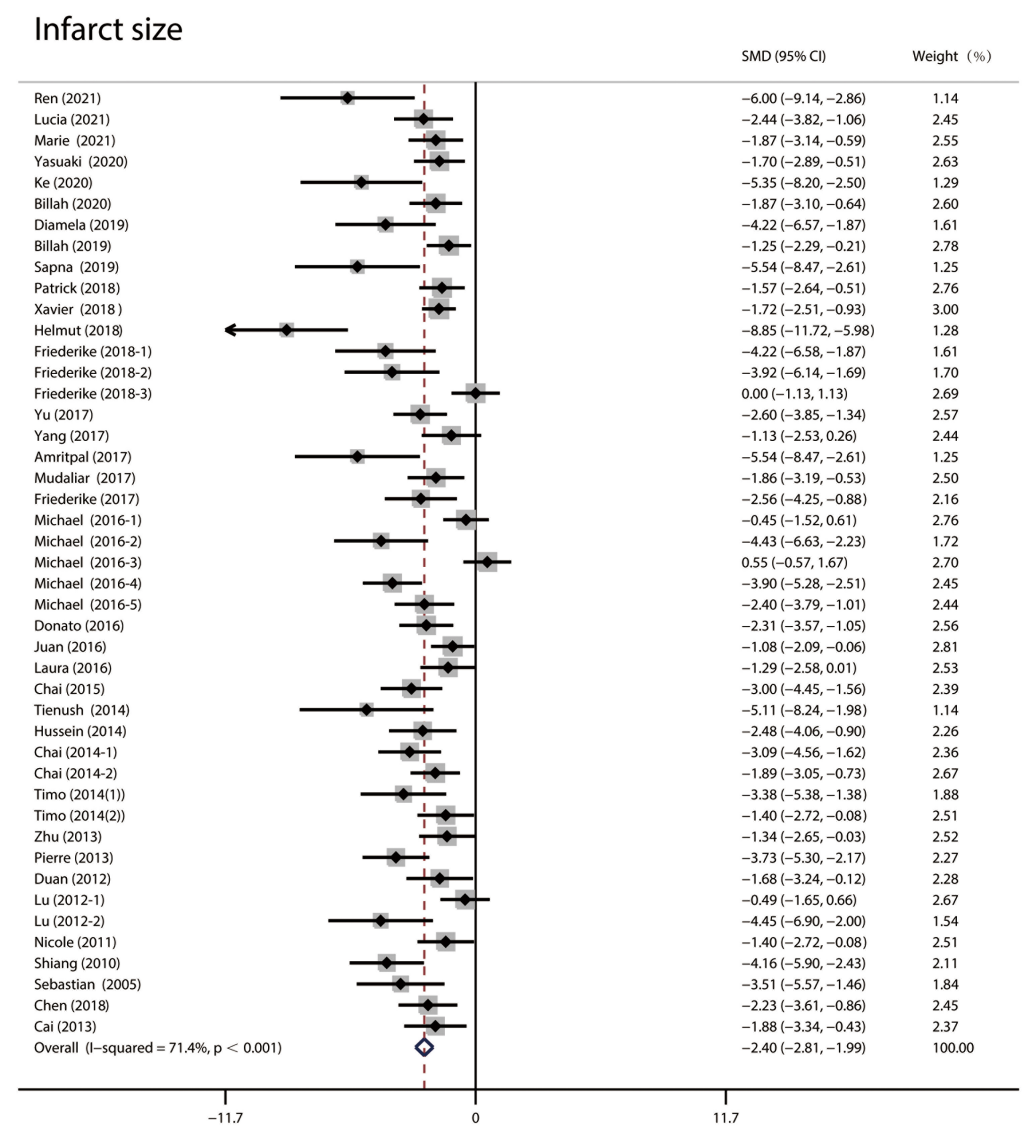
**Forest plots of infarct size in random effect size analysis**. High heterogeneity was observed, and the infarct size in the RIPC group was significantly lower than in the control group. RIPC, remote Ischemic preconditioning; CI, confidence interval; SMD, standardized mean difference.

The infarct size was analyzed using random effect size because of the high heterogeneity ($I^{2}$ = 71.4%). As shown in Fig. 2, the infarct size in the RIPC group was significantly smaller than that in the control group (standardized mean difference (SMD): –2.40; 95% CI: –2.81, –1.99; *p *< 0.001).

Subgroup analysis was performed based on species, study type, anesthesia method, reperfusion time, staining method, RIPC site, duration, and cycles (Table 3). The difference between the RIPC and control groups was significant in most subgroups (*p *< 0.01). However, there was no significant difference between the RIPC and control groups when the RIPC site was the hepatic vessel (SMD: –3.354; 95% CI: –8.103, 1.395; *p* = 0.166) and infrarenal aorta (SMD: –1.216; 95% CI: –3.411, 0.978; *p* = 0.277). In addition, subgroup analysis of RIPC duration yielded different results; no significant difference was observed between the 5/15 min group (SMD: –0.451; 95% CI: –1.517, 0.615; *p* = 0.407) and 30/15 min group (SMD: 0.548; 95% CI: –0.571, 1.668; *p* = 0.337). In the analysis of the different staining methods, the heterogeneity decreased from 71.4% to 59%, 69.2%, and 65.3%, indicating that the staining method might be a source of heterogeneity.

**Table 3.** **Subgroup analysis**.

Subgroup		Number	SMD	95% CI		Weight %	*p* value	Heterogeneity
Species								
	Rats	39	–2.328	–2.775	–1.881	87.77	<0.001	72.20%
	Mice	6	–2.983	–4.145	–1.82	12.23	<0.001	67.10%
Anesthesia								
	Inhalation	4	–2.005	–2.878	–1.131	9.54	<0.001	38.60%
	Injection	40	–2.473	–2.935	–2.011	87.96	<0.001	73.80%
	Unclear	1	–1.86	–3.186	–0.534	2.5	0.006	NA
I/R method								
	*Ex vivo*	10	–3.638	–4.765	–2.512	18.94	<0.001	73.60%
	*In vivo*	35	–2.118	–2.539	–1.697	81.06	<0.001	68.10%
Reperfusion duration								
	1–1.5 h	8	–2.368	–3.701	–1.036	18.05	<0.001	84.90%
	2–3 h	33	–2.462	–2.924	–2.001	72.92	<0.001	68.10%
	24 h	4	–1.911	–2.863	–0.958	9.02	<0.001	44.10%
RIPC site								
	Limb	22	–2.462	–3.046	–1.879	47.64	<0.001	70.00%
	Hepatic vessel	2	–3.354	–8.103	1.395	3.58	0.166	87.00%
	Femoral artery	15	–2.445	–3.055	–1.834	34.06	<0.001	59.60%
	Infrarenal aorta	3	–1.216	–3.411	0.978	7.18	0.277	87.20%
	Mesenteric artery	2	–3.151	–4.621	–1.681	4.88	<0.001	55.30%
	Abdominal aorta	1	–1.891	–3.049	–0.732	2.67	0.001	NA
RIPC duration								
	5/5 min	38	–2.365	–2.775	–1.954	83.83	<0.001	65.40%
	5/15 min	1	–0.451	–1.517	0.615	2.76	0.407	NA
	10/10 min	1	–3.734	–5.297	–2.171	2.27	<0.001	NA
	15/15 min	4	–3.404	–4.279	–2.528	8.44	<0.001	9.90%
	30/15 min	1	0.548	–0.571	1.668	2.7	0.337	NA
RIPC cycles								
	1 cycle	8	–2.177	–3.51	–0.844	18.84	0.001	86.20%
	3 cycles	22	–2.474	–2.98	–1.969	49.54	<0.001	62.20%
	4 cycles	15	–2.356	–3.073	–1.638	31.63	<0.001	67.30%
Staining method								
	TTC	12	–2.556	–3.287	–1.825	25.06	<0.001	59.00%
	TTC-Evans blue	30	–2.095	–2.549	–1.64	70.53	<0.001	69.20%
	TTC-patent blue	2	–7.098	–10.528	–3.668	2.57	<0.001	65.30%
	TTC-black Chinese ink	1	–3.515	–5.57	–1.459	1.84	0.001	NA

Abbreviation: RIPC, remote Ischemic preconditioning; I/R, ischemia/reperfusion; TTC, 2,3,5-Triphenyltetrazolium chloride; SMD, standardized mean difference; CI, confidence interval; NA, not available.

Meta-regression analysis was performed to detect any possible sources of heterogeneity (Table 4). Heterogeneity factors included species, I/R method, anesthesia, reperfusion time, staining method, RIPC site, duration, and cycles. The results indicated that the study type (*p* = 0.001) and staining method (*p* = 0.013) might be sources of heterogeneity.

**Table 4.** **Meta regression analysis**.

Factor	Coefficient	Std. Err	t	*p* value	95% CI
Species	1.060412	0.7194901	1.47	0.149	–0.3987814, 2.519606
Anesthesia	0.278913	0.6531675	0.43	0.672	–1.045772, 1.603598
I/R method	–2.80989	0.7620935	–3.69	0.001	–4.355487, –1.264293
Reperfusion duration	–0.5447279	0.6522578	–0.84	0.409	–1.867568, 0.7781122
RIPC site	–0.0570877	0.2852199	–0.20	0.842	–0.6355406, 0.5213651
RIPC duration	–0.2409839	0.3198366	–0.75	0.456	–0.8896425, 0.4076747
RIPC cycles	–0.5557973	0.5625913	–0.99	0.330	–1.696785, 0.5851908
Staining method	–1.331171	0.509424	–2.61	0.013	–2.36433, –0.298011

Abbreviation: RIPC, remote Ischemic preconditioning; I/R, ischemia/reperfusion; CI, confidence interval; Std. Err, standard error.

**Fig. 3.**
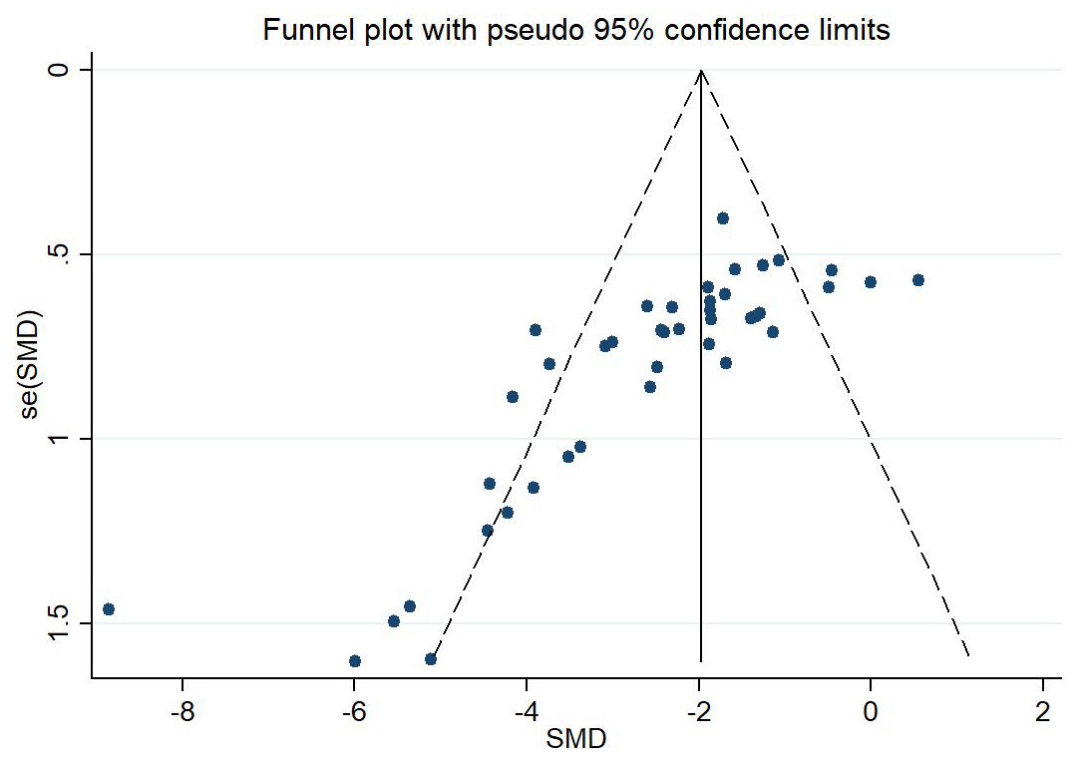
**Assessment of publication bias by Funnel plot**. The dotted line indicated the standardized mean difference (SMD). The funnel plot was unsymmetric and indicated the existence of publication bias. SMD, standardized mean difference.

The funnel plot was unsymmetric and the Egger’s test (*p *< 0.001) and Begg’s test (*p *< 0.001) confirmed the publication bias (Fig. 3).

**Fig. 4.**
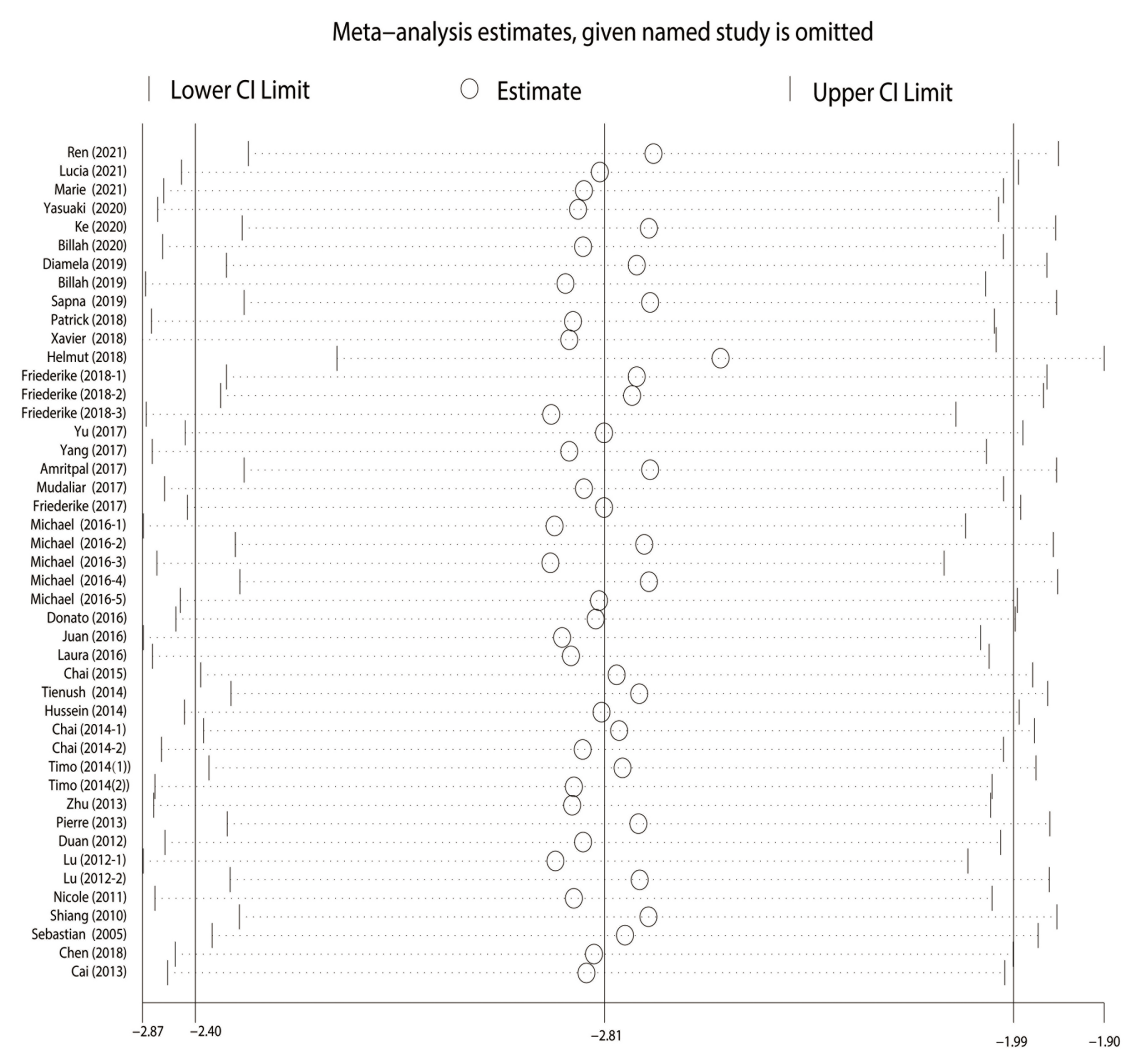
**Sensitivity analysis**. The round shape represented the estimated pooled effect when a given named study was omitted. The vertical line indicated the lower and upper confidence interval (CI) limit.

The nonparametric trim-and-fill method was applied to adjust the effect size, and the results were robust (*p *< 0.001). Sensitivity analysis also concluded that the results were robust in our study (Fig. 4).

#### 3.4.2 Serum Cardiac Markers

**Fig. 5.**
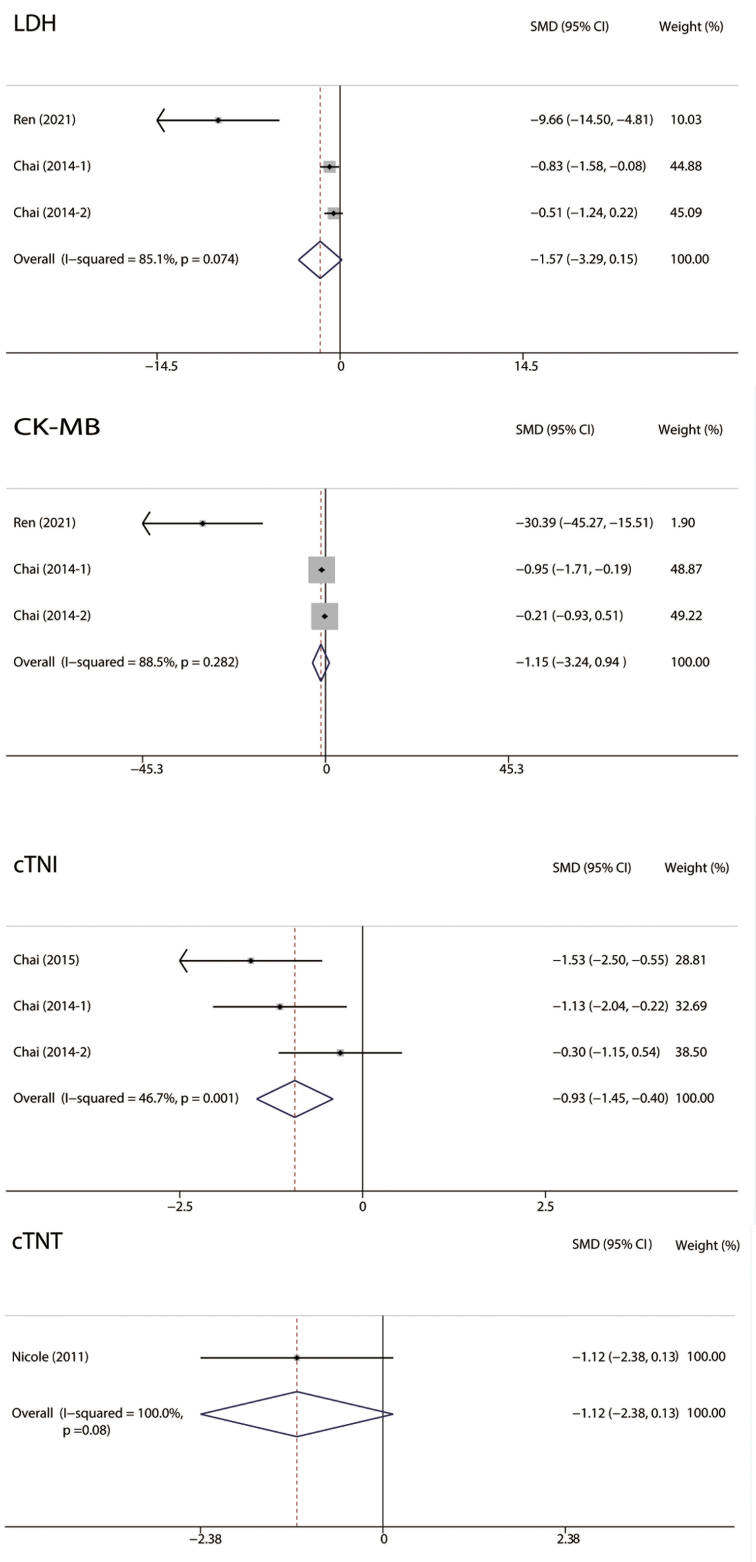
**Forest plots of Serum cardiac markers**. RIPC was related to significantly lower cTNI levels in MIRI. No significant difference was observed in LDH, CK-MB, and cTNT between the two groups. RIPC, remote Ischemic preconditioning; MIRI, myocardial ischemia-reperfusion injury; CI, confidence interval; CK, creatine kinase; cTNI, cardiac troponin I; cTNT, cardiac troponin T; LDH, lactate dehydrogenase; SMD, standardized mean difference.

The levels of serum cardiac markers, including lactic dehydrogenase (LDH), creatine kinase-MB (CK-MB), cardiac troponin I (cTNI), and cardiac troponin T (cTNT), were analyzed (Fig. 5). Studies reporting cTNI levels showed low heterogeneity ($I^{2}$ = 46.7%). They suggested that RIPC was associated with significantly lower cTNI levels in MIRI (SMD: –0.93; 95% CI: –1.45, –0.4; *p* = 0.001). There was no significant difference in LDH ($I^{2}$ = 85.1%; SMD: –1.57; 95% CI: –3.29, 0.15; *p* = 0.074), CK-MB ($I^{2}$ = 88.5%; SMD: –1.15; 95% CI: –3.24, 0.94; *p* = 0.282), and cTNT (SMD: –1.12; 95% CI: –2.38, 0.13; *p* = 0.08) levels between the RIPC and control groups.

#### 3.4.3 Vital Signs

**Fig. 6.**
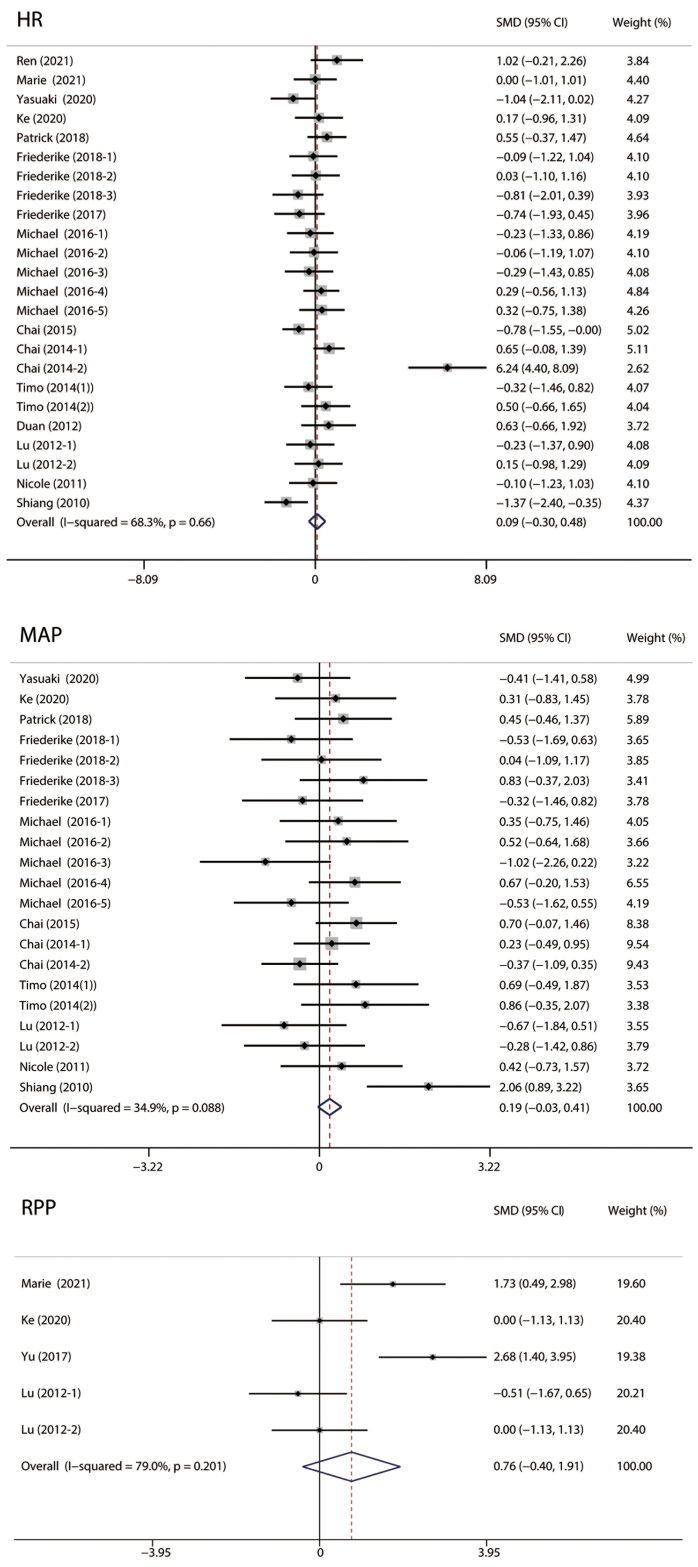
**Forest plots of vital signs**. No significant difference was observed in HR, MAP and RPP. HR, heart rate; MAP, mean arterial pressure; RPP, heart rate-blood pressure product.

As shown in Fig. 6, we analyzed the vital signs of MIRI animals, including heart rate (HR), mean arterial pressure (MAP), and rate pressure product (RPP). No significant difference was observed in HR ($I^{2}$ = 68.3%; SMD: 0.09; 95% CI: –0.3, 0.48; *p* = 0.66), MAP ($I^{2}$ = 34.9%; SMD: 0.19; 95% CI: –0.03, 0.41; *p* = 0.088) and RPP ($I^{2}$ = 79.0%; SMD: 0.76; 95% CI: –0.40, 1.91; *p* = 0.201), suggesting that RIPC did not improve vital signs in MIRI animals.

#### 3.4.4 Hemodynamic Parameters

**Fig. 7.**
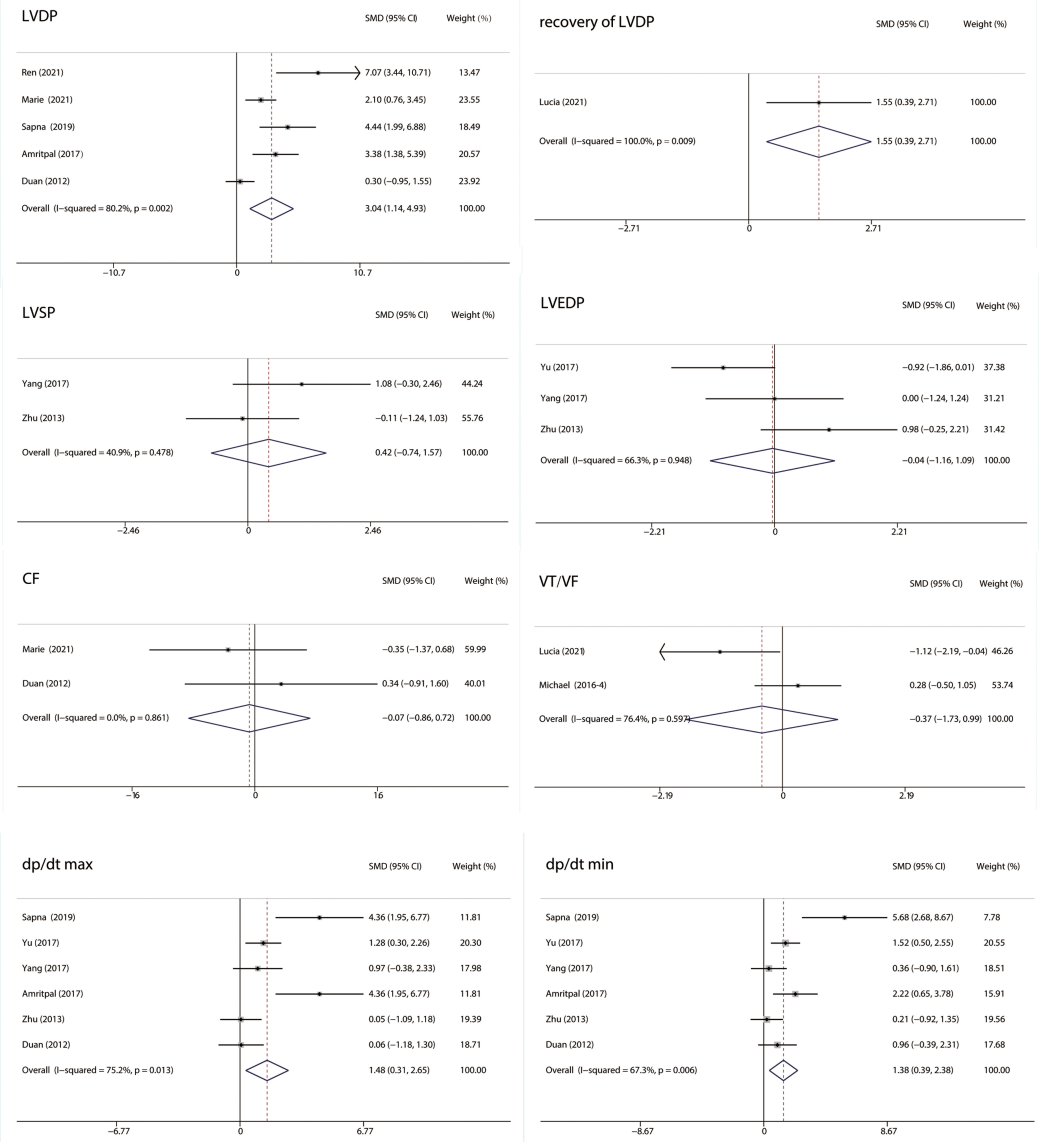
**Forest plots of hemodynamic parameters**. LVDP, recovery of LVDP, dp/dt max, and dp/dt min were significantly higher in the RIPC group. No significant difference was observed in LVSP, LVEDP, CF, and VT/VF between the two groups. RIPC, remote Ischemic preconditioning; CI, confidence interval; SMD, standardized mean difference; CF, coronary flow; LVDP, left ventricular developed pressure; LVEDP, left ventricular end diastolic pressure; LVSP, left ventricular systolic pressure; VT, ventricular tachycardia; VF, ventricular fibrillation.

Left ventricular developed pressure (LVDP), recovery of LVDP, left ventricular systolic pressure (LVSP), left ventricular end-diastolic pressure (LVEDP), coronary flow (CF), ventricular tachycardia (VT), ventricular fibrillation (VF), dp/dt max and dp/dt min were included in the comparison of hemodynamic parameters between the RIPC and control groups (Fig. 7). LVDP ($I^{2}$ = 80.2%; SMD: 3.04; 95% CI: 1.14, 4.93; *p* = 0.002), LVDP recovery (SMD: 1.55; 95% CI: 0.39, 2.71; *p* = 0.009), dp/dt max ($I^{2}$ = 75.2%; SMD: 1.48; 95% CI: 0.31, 2.65; *p* = 0.013) and dp/dt min ($I^{2}$ = 67.3%; SMD:1.38; 95% CI: 0.39, 2.38; *p* = 0.006) were significantly higher in the RIPC group. No significant differences were observed in LVSP ($I^{2}$ = 40.9%; SMD: 0.42; 95% CI: –0.74, 1.57; *p* = 0.478), LVEDP ($I^{2}$ = 66.3%; SMD: –0.04; 95% CI: –1.16, 1.09; *p* = 0.948), CF ($I^{2}$ = 0%; SMD: –0.07; 95% CI: –0.86, 0.72; *p* = 0.861) and VT/VF ($I^{2}$ = 76.4%; SMD: –0.37; 95% CI: –1.73, 0.99; *p* = 0.597) between the RIPC and control groups in MIRI animals.

#### 3.4.5 Cell Death

**Fig. 8.**
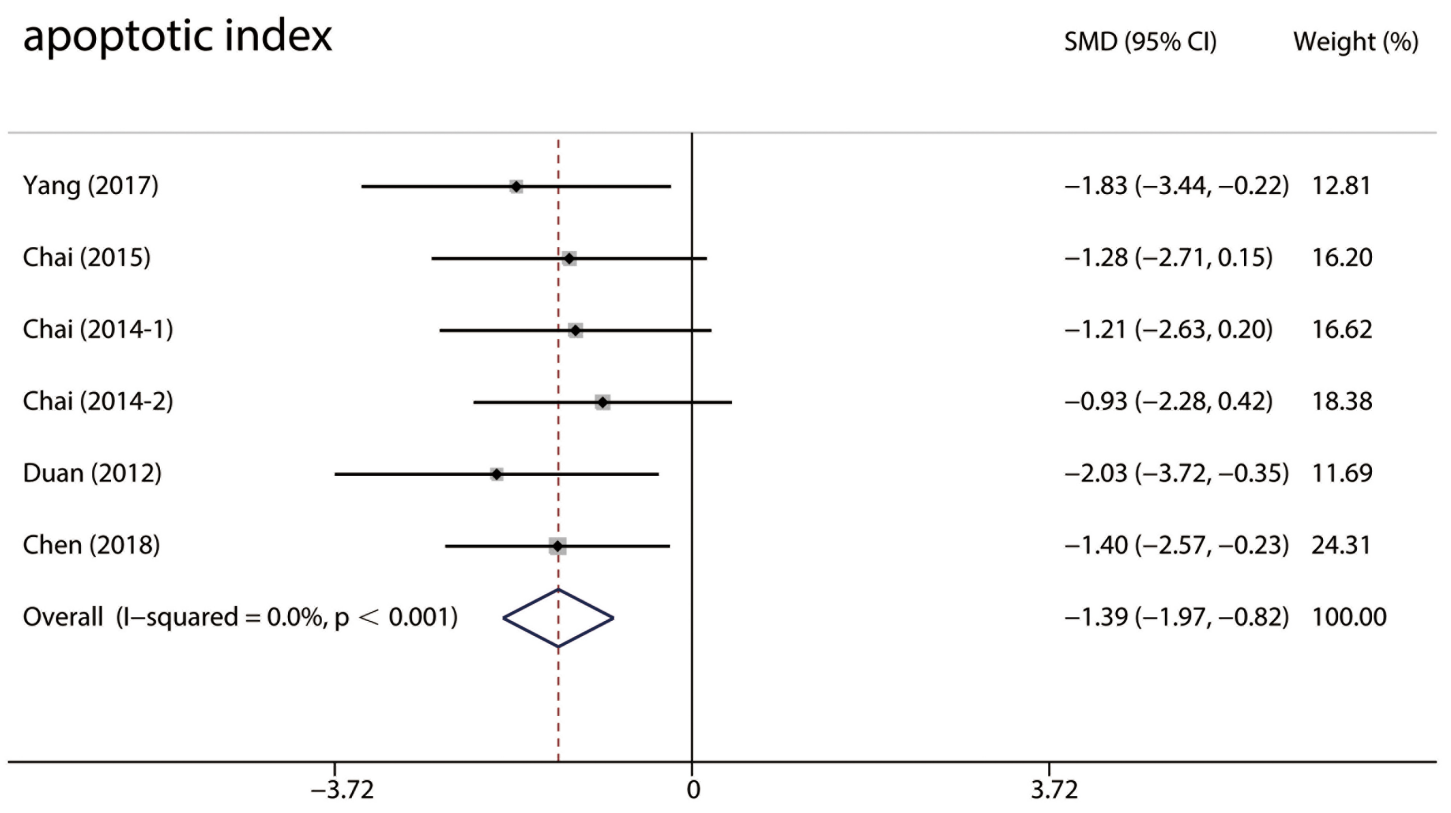
**Forest plots of the TUNEL-positive cells**. TUNEL-positive cells in the RIPC group were significantly less compared with the control group in MIRI animals. RIPC, remote Ischemic preconditioning; CI, confidence interval; MIRI, myocardial ischemia-reperfusion injury; SMD, standardized mean difference.

Six studies [26, 29, 37, 40, 47] used the terminal deoxynucleotidyl transferase dUTP nick end labeling (TUNEL) method to identify cell death and no heterogeneity was observed ($I^{2}$ = 0%). A fixed-effects model was applied to the pooled effect estimates which indicated significantly fewer TUNEL-positive cells in the RIPC group than in the control group (SMD: –1.39; 95% CI: –1.97, –0.82; *p *< 0.001) (Fig. 8).

## 4. Discussion

This systematic review and meta-analysis included 713 male animals from 37 studies. The infarct size was considered the primary outcome, and all the studies’ data were complete. Forest plots, subgroup analysis, sensitivity analysis, meta-regression, funnel plot, Egger’s test, and Begg’s test were performed. This study found that RIPC significantly protected against MIRI in rats and mice by reducing infarct size, decreasing serum myocardial markers and the number of TUNEL-positive cells, and improving cardiac function. Furthermore, we also provide evidence regarding the factors influencing RIPC, such as the cycles, duration, and site of RIPC, which might be helpful in the clinical setting.

RIPC is expected to benefit the progression of cardiovascular disease as a noninvasive and effective treatment. The mechanism of cardioprotection induced by RIPC contains two phases. The early phase follows the RIPC stimulus and lasts for 1–2 h, while the delayed phase appears 12–24 h later and lasts for 48–72 h [51]. In the early phase, RIPC protects against MIRI via humoral, neuronal, and systemic pathways [12]. The signaling pathways, including the reperfusion injury salvage kinase pathway and the survival activating factor enhancement pathway, are involved in protecting the myocardium [52]. In the delayed phase, RIPC protects against MIRI by downregulating the oxidative and inflammatory injury gene expression, and the mTOR signaling, whereas enhancing the autophagy signaling [12, 53, 54]. Redox stress is related to inflammasomes [55] such as Nlrp3, which can activate caspase-1, damage the cell membrane, cause pyroptosis, and contribute to MIRI [56, 57, 58]. The balance of redox reactions, including that between reactive oxygen and nitrogen species, plays an important role in MIRI and participates in cardioprotection from preconditioning and postconditioning [59] by a specific posttranslational modification (S-nitrosylation of proteins) [60, 61]. In addition, it is emerging that inflammation plays an important role in long-term cardioprotective effects, including cardiac remodeling and heart failure, which has been neglected in previous studies [62].

Primary outcome analysis using Forest plots suggested that RIPC significantly reduced infarct size, similar to that reported in a previous systematic review and meta-analysis that included data from 653 animals and analyzed the effect of remote ischemic conditioning (RIC) in *in vivo* animal models of MIRI [63]. In the analysis of secondary outcomes, only RIPC significantly reduced the level of cTNI, which might be because cTNI has higher sensitivity and specificity for myocardial injury than LDH, CK-MB, and cTNT [64, 65]. We also found higher LVDP, recovery of LVDP, dp/dt max, and dp/dt min in the RIPC group, representing better left ventricular diastolic function. Another study also suggested that LVEDP and LVDP were associated with reduced infarct size in RIC, which is consistent with our results [54]. In addition, the TUNEL-positive cells were significantly fewer in the RIPC group. However, TUNEL staining may indicate cell apoptosis or necroptosis [66]. The major cell death contributing to MIRI includes apoptosis or necroptosis in non-cardiomyocytes and necroptosis or pyroptosis in cardiomyocytes [5]. Contribution of apoptosis in cardiomyocytes in MIRI is controversial. Inserte *et al*. [67] suggested that caspase-mediated apoptosis does not significantly contribute to infarct size and ventricular remodeling in MIRI. Therefore, TUNEL-positive cells do not only refer to apoptosis; other cell death mechanisms that contribute to MIRI, such as necroptosis and pyroptosis, also need to be considered in further study. Furthermore, no significant differences in vital signs were observed between the two groups. This might be because a high HR can maintain blood pressure in rats and mice.

In the subgroup analysis, RIPC in the hepatic vessel and infrarenal aorta groups did not show a significant protective effect, indicating that cardioprotection depends on RIPC sites. Different mechanisms, including humoral, neuronal, or systemic pathways, might be related to different organs subjected to RIPC, which could explain this phenomenon [33]. A previous study indicated that the number and duration of RIPC cycles determine the efficacy of the RIPC [68]. This study observed an interesting phenomenon: compared with the same RIPC duration of ischemia and reperfusion (5/5 min, 10/10 min, 15/15 min), RIPC durations of 5/15 min and 30/15 min did not show a significant reduction in infarct size in MIRI. Based on the hypothesis of Galagudza *et al*. [33], the duration of ischemia and reperfusion might influence the balance between the accumulation of metabolites and/or signaling molecules and the washout of these signaling agents, which influence the triggers of the cardioprotective response in the heart. Therefore, the different durations of ischemia and reperfusion might have disrupted the balance and reduced RIPC cardioprotection in the 5/15 min and 30/15 min groups.

The subgroup analysis and meta-regression results indicated that the possible source of heterogeneity was the I/R method and staining method. The I/R method included *in vivo* and *ex vivo* studies; therefore, the systemic response that relies on circulation, such as inflammatory reaction in MIRI, might cause heterogeneity among studies. The staining methods of MIRI included staining with TTC and TTC-Evans blue/patent blue/black Chinese ink, which could lead to different sizes of the area at risk (AAR) and influence the result of infarct size/AAR, resulting in high heterogeneity. As for species, rats have lesser collateral blood flow and faster infarct progression than mice, which might influence the infarct size, but it was not a possible source of heterogeneity in this study [69]. Despite similarities in the cycles and duration, the results can still differ in terms of the efficacy of RIPC in MIRI. In a previous systematic review and meta-analysis, Bromage *et al*. [63] reported significant heterogeneity that could not be explained by any of the experimental variables analyzed by meta-regression. Recently, Penna *et al*. [70] reported that keeping the ischemic conditioned limb warm (40 °C) can increase the cardioprotective efficacy of RIPC, indicating that limb temperature could be a potential source of heterogeneity that was not considered in the report by Bromage *et al*. [63] and this meta-analysis because of missing data.

The funnel plot, Egger’s test, and Begg’s test suggested the existence of publication bias, which may be because some studies did not report negative results. However, the sensitivity analysis and the nonparametric trim-and-fill method showed that the overall effect of RIPC on infarct size in MIRI was robust. The quality of the included studies varied, mainly because of the differences in random housing and blinding methods.

In a multicenter, randomized controlled trial, RIPC did not show a relevant benefit for cardiac surgery, which was different from preliminary experience in animals [8]. The discrepancies between animal studies and clinical studies contains the age of patients and animals, RIPC site, and outcome measures. In this clinical study, the mean age of patients was 65.8 years, the RIPC was induced in upper limbs, and the outcome measures included a composite of death, myocardial infarction, stroke, or acute renal failure. In animal studies, researchers often choose the hind limbs as the RIPC site and measured the infarct size in young mice and rats. The age was considered as one of the operative mortality risk factors [71], which might influence the results. In addition, the nervous system takes part in protective effect of RIPC [25]. Therefore, differences in the neural pathways of the upper and hind limbs may affect the efficacy of RIPC. Furthermore, the outcome measures in clinical study were more complicated than in animal studies, which might lead to controversial results.

This study had several limitations, which should be mentioned. First, evaluation might be influenced by high heterogeneity owing to differences between animal experiments; better-performed studies with particular emphasis on the detailed characterization of RIPC protocols and analysis are warranted. In addition, we did not include studies with female animals because endogenous estrogen was suggested to limit cardiomyocyte apoptosis from MIRI by producing a baseline anti-apoptotic profile; further studies could focus on the potential effect of sex [72]. Moreover, only studies in rats and mice were analyzed. The potential benefits of RIPC in larger species and the presence of comorbidities need to be investigated before studies in humans.

## 5. Conclusions

This systematic review and meta-analysis analyzed the protective effect of RIPC against MIRI. It was found that RIPC significantly protected against MIRI in small animal models by reducing infarct size, decreasing serum myocardial markers and limiting cell death, and improving cardiac function. RIPC duration and site influence the protective effect of RIPC on MIRI, which contributes in reducing confounding factors and determines the best approach for human studies.

## Supplementary Material

Supplementary material associated with this article can be found, in the online version, at https://doi.org/10.31083/j.rcm2312413.
